# Hybrid stent in management of malignant airway obstruction with carina esophageal fistula: A case report

**DOI:** 10.1097/MD.0000000000033405

**Published:** 2022-04-07

**Authors:** Chenghua Zhu, Jingning Liu, Mingyao Ke, Yazhi Yong, Bingqing Luo, Ganzhu Feng

**Affiliations:** a Department of Respiratory and Critical Care Medicine, Nanjing Medical University Second Affiliated Hospital, Nanjing, Jiangsu, China; b Department Respiratory and Critical Care Medicine, Xiamen Medical College Second Affiliated Hospital, Xiamen, Fujian, China.

**Keywords:** hybrid stent, malignant central airway obstruction, trachea carina and esophageal fistula

## Abstract

Airway stents have been developed rapidly to treat airway stenosis and fistula caused by various reasons. Malignant conditions that lead to central airway obstruction, especially the invasion of trachea carina and formation of esophageal fistula, are still a challenge for clinicians.

**Patient concerns:**

A 61-year-old man presented with malignant airway obstruction and fistula between trachea carina and esophagus accompanied by severe respiratory failure.

**Diagnosis:**

The patient was clinically diagnosed with esophageal squamous cell cancer of stage IV, carina esophageal fistula, severe pneumonia, hypoproteinemia.

**Interventions:**

Y-shaped covered metallic stent and Y-type silicone stent (hybrid stent) were placed in the airway to increase tracheal patency, block the fistula and perform carinal plasty.

**Outcomes:**

The clinical symptoms of the patient improved rapidly and the lung infection was controlled effectively. This patient was followed up for more than 2 month, and the quality of life was better than before.

**Lessons:**

Hybrid stent can be used as 1 of options for airway reconstruction and palliative treatment for patients with complex airway diseases caused by malignant tumors.

## 1. Introduction

Malignant central airway obstruction (MCAO) is common in advanced-stage lung cancers and pulmonary metastatic carcinomas.^[1]^ Nonsurgical techniques, such as laser photocoagulation, rigid bronchoscopic dilatation, and endobronchial stent placement are currently employed for palliation of intrinsic obstruction of the airways.^[2]^

Airway stents placement is often performed to avoid airway obstruction by preventing malignant tumor compression, which is a palliative treatment for MCAO, to relieve symptoms of respiratory distress, prolong survival time, and improve lung function.^[3]^ There is always a dispute about which kinds of stent is better used to treat MCAO diseases.^[2,4]^ Patients with MCAO involving trachea carina infiltration, or fistula formation are more difficult to treat, and clinical data are not enough at present.

We herein report a case of Y type covered metallic stent combined with Y type silicone stent in the management of MCAO involving trachea carina esophageal fistula, which provides a clinical basis for the treatment of complex airway diseases.

### 1.1. Clinical data

**Figure 1.**
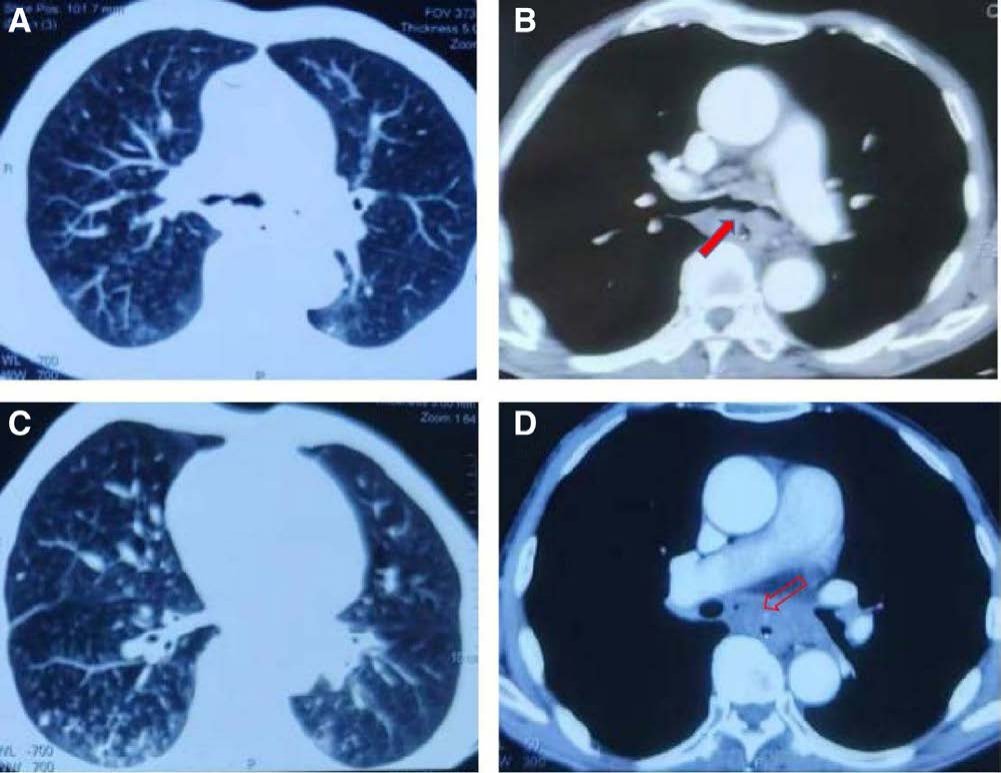
Chest enhanced CT scan of this 61-year-old male patient showed the esophageal fistula involved trachea carina as indicated by the red arrow, and enlarged mediastinal lymph nodes as showed by the red hollow arrow. CT = computed tomography.

A 61-year-old male patient was admitted to the Department of Intensive Care Unit of Respiratory and Critical Care Medicine in the Second Affiliated Hospital of Xiamen Medical College on September 19 in 2022. The patient presented with cough, expectoration and asthma for more than 1 month after the surgery of esophageal squamous cell carcinoma. The patient was diagnosed as esophageal squamous cell carcinoma in the other hospital 1 year ago, and received neoadjuvant therapy with 2 cycles of chemotherapy and immunotherapy. After surgery, the patient was treated with 4 cycles of chemotherapy and immunotherapy, followed by a course of radiotherapy. The patient developed symptoms of cough with moderate amounts of purulent sputum and asthma, which was significantly aggravated after mild activity. On August 4 in 2022, the fibroptic bronchoscopy of the patient in the local hospital revealed that there was large amount of purulent secretions in the tracheal cavity, and a fistula between trachea carina and esophagus with vascular fluctuations on the surface. NGS of alveolar lavage fluid indicated infection with Acinetobacter baumannii and Candida tropicalis. Computed tomography of the chest on September 11 showed postesophagectomy status, anastomotic stoma slightly thickened, trachea carina fistula, bilateral pneumonia, mediastinal lymph node enlargement, and metastasis considered (Fig. 1). The patient was diagnosed as “carina esophageal fistula, esophageal squamous cell carcinoma of stage IV, severe pneumonia, hypoproteinemia.” He also has a medical history of hypertension and lumbar fracture.

### 1.2. Physical examination

The patient general condition was bad. Vital signs were as follows: temperature was 36.9°C, heart rate was 155 beats per minute, respiratory rate was 26 times per minute, and blood pressure was 138/88 mm Hg. The patient was tachypnea and looked sick with cyanosis of lips. Wet rales were heard in the bilateral lungs, and no swelling was seen in both lower limbs.

### 1.3. Laboratory investigation

After hospitalization, the patient was immediately treated with electrocardiographic monitor and noninvasive ventilator to assist respiration [Bi-level positive airway pressure mode, fraction of inspiration O_2_ 50%, inspired positive airway pressure 12 cmH_2_O, and expired positive airway pressure 4 cmH_2_O]. the patient arterial blood gas was pH 7.47, PCO_2_ 54.9 mm Hg, PO_2_ 78.2 mm Hg (noninvasive ventilator, fraction of inspiration O2 50%); blood routine was white blood cell 14.72 × 10^9^/L, neutral 92.5%, hemoglobin 78 g/L; biochemistry was blood urea nitrogen 10.1 mmol/L, creatinine 49 μmol/L, procalcitonin 0.98 ng/L; coagulation routine was activated partial thromboplastin time 39.1 seconds, prothrombin time 18.4 seconds, D-Dimer 527 ng/mL; and electrocardiogram was atrial flutter.

### 1.4. Treatment

**Figure 2.**
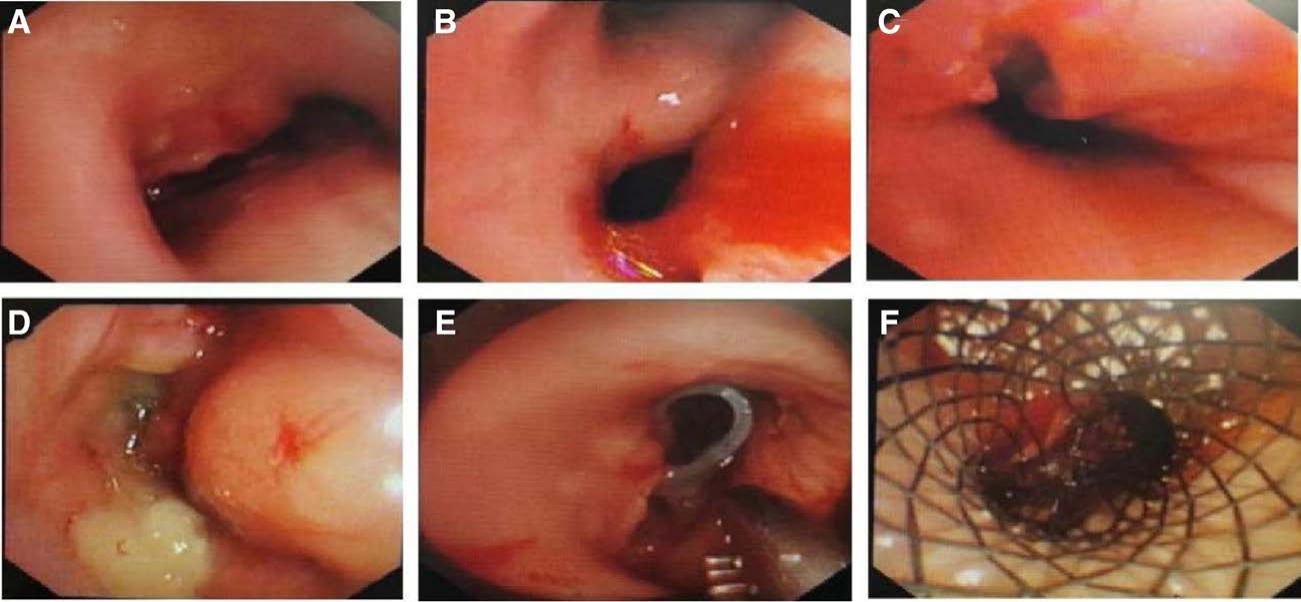
Rigid tracheoscopic view of the trachea and stents implantation. A: trachea carina disappeared, B: right main bronchus involved by fistula, C: left main bronchus infiltrated and narrowed, D: a fistula between trachea carina and esophagus, E: a Y-shaped Dumon placed stent in the right main bronchus, F: a Y-shaped metal covered stent placed in the lower trachea.

Antibiotic drugs imipenem cilastatin and tegacycline were administered intravenously and enteral nutrition was administered through jejunal nutrition tube. In the morning of September 20, under general anesthesia, a large amount of purulent secretions in the trachea was seen by placing a rigid tracheoscope through the mouth of the patient. Tracheal carina was disappeared, a large fistula was seen behind the location of carina and extended to the right main bronchus with about 0.5 cm from the right intermediate bronchus, and the left main bronchus was obviously infiltrated and narrowed. A Y-shaped silicone stent was placed in the right main bronchus (model: main branch 15 × 18 mm, side branches 12 × 20 mm and 12 × 8 mm), and a Y-shaped covered metallic stent (model: trachea section 16 × 40 mm, right branch 14 × 15 mm, left branch 12 × 30 mm) was placed in the lower part of trachea (Fig. 2) after attracting sputum.

The patient was sent to respiratory intensive care unit (RICU) for resuscitation after operation. Postoperative examination was performed. Arterial blood gas results: PH 7.45, PCO_2_ 48.2 mm Hg, PO_2_ 73.4 mm Hg (nasal catheter oxygen inhalation 3 L/min), and electrocardiogram showed sinus rhythm, short PR interval. In the afternoon of September 20 and September 21, bedside bronchoscopy was performed and showed that the stents were unobstructed and the purulent secretions was less than before. Two days later, the asthma symptoms of the patient improved, and oxygen saturation (SpO_2_) was about 94% by inhaling oxygen for 3 L/min. The sputum volume decreased, and oxygenation index improved. The laboratory test results of white cell count, C-reactive protein (CRP), blood urea nitrogen, albumin and arterial blood gas were improved 9 days after operation (Table 1). The antiinfection treatment was changed into piperacillin tazobactam combined with levofloxacin based on the culture result of alveolar lavage fluid was Pseudomonas aeruginosa.

**Table 1:** Laboratory inspection results for venous blood and arterial blood before and after stents placement.

Variable	Result (before operation)	Result (9 d after operation)
White-cell count (per mm^3^)	14720	10700
Hemoglobin (g/dL)	7.8	6.9
C-reactive protein (mg/dL)	40.51	29.95
Blood urea nitrogen (mmol/L)	10.1	3.21
Albumin (g/L)	34.14	35.05
pH	7.47	7.45
PCO_2_ (mm Hg)	54.9	48.2
PO_2_/FIO_2_ (mm Hg)	156.4	222.4

FIO2 = fraction of inspiration O2.

## 2. Discussion

The first Y-stent was a silastic stent developed in 1972 by Neville and colleagues.^[5]^ Throughout the following years, airway stents were further improved. Nowadays, different stents that differ in material, rigidity, and insertion technique are available. The most commonly used stents are silicone stents and metal stents. The advantages of silicone stents include: easy to form new granulation tissue, and easy to be removed. Disadvantages are as follows: poor shape-adaptability, secretion retention, easy migration, and implantation difficultly. Advantages of metal stents include: strong support, not easy to shift, It can be easily implanted under local anesthesia. Disadvantages are: granulation tissue formation, difficulty in taking out.^[6]^ Selection of a stent usually depends on whether the patient has a benign or malignant disease, the stent is to be used temporarily or permanently, experience of the physician with stent insertion, and medical cost for the procedure.^[7]^

MCAO is a life-threatening disease,^[8]^ in which malignant carinal stenosis is the most severe scenario. The causes of MCAO include lung cancer and other tumors, such as thyroid cancer, thymoma, thymus carcinoma, mediastinal germ cell cancer or metastases, which might promote external compression. Malignant pleural mesothelioma and esophageal carcinoma additionally tend to present an infiltrative or destructive growth with endobronchial tumor infiltration and/or fistula formation.^[9]^ MCAO is often accompanied by infections such as tuberculosis, fungus and virus. The treatment indications should be discussed by a multidisciplinary team of medical experts.^[10]^ However, stents implantation is mostly performed by well-trained interventional pulmonary specialists.^[11]^ Which type of stents to choose still remains controversial. Dutau H, et al found that silicone stent placement improved dyspnea symptoms more significantly among 170 patients who were under symptomatic airway obstruction due to non-small cell lung cancer, and lasted longer, but did not change the survival. Stent placement was not suggested in patients without previous oncologic treatment.^[12]^ Oki M, et al found stent placement with new dedicated bifurcated silicone stent designed to fit on the primary right carina was feasible, effective, and acceptably safe.^[13]^ Wang Y, et al found the placement of radioactive bare metal stent in patients with inoperable malignant airway obstruction to be safe, and it significantly reduced restenosis and improved overall survival compared with placement of conventional bare metal stent.^[14]^ Kim JH, et al suggested the use of a retrievable metallic stent internally coated with silicone was safe and effective method for relieving dyspnea, with adequate stent patency in patients with benign or MCAOs, but there were some complications (31%) after stent placement, including tumor overgrowth, stent migration, symptomatic granulation tissue formation, and esophagobronchial fistula.^[15]^ Many studies have shown that, the median overall survival time for central airway obstruction caused by esophageal cancer was significantly shorter than that caused by lung cancer,^[9,16]^ which was about 60 days for patients with esophageal cancer and about 56 days for patients with esophagotracheal fistula.^9^ Ortiz-Comino RM, et al retrospectively analyzed the efficacy and complications after placement of a full covered self-expandable metal stent (Aerstent) and a silicone stent (Dumon), and both stents were equally successful and safe.^[17]^ A retrospective analysis of 36 patients with malignant protuberance lesions showed that, bare Y metal stents could be used as a bridging method before commencing adjuvant therapy and final palliative therapy for relief of symptoms. The complications include restenosis, stent rupture, and esophago tracheal fistula.^[4]^ Malignant esophagotracheal fistula is a fatal complication of esophageal or bronchial cancer, which may be related to cancer progression and cancer treatment. Malignant protuberant mediastinal fistula is particularly rare, with poor prognosis. The purpose of the treatment is only to improve the quality of life, reduce the incidence of aspiration and sepsis. The common treatment method is to insert covered metal stent or silicone stent into the trachea.^[18]^

**Figure 3.**
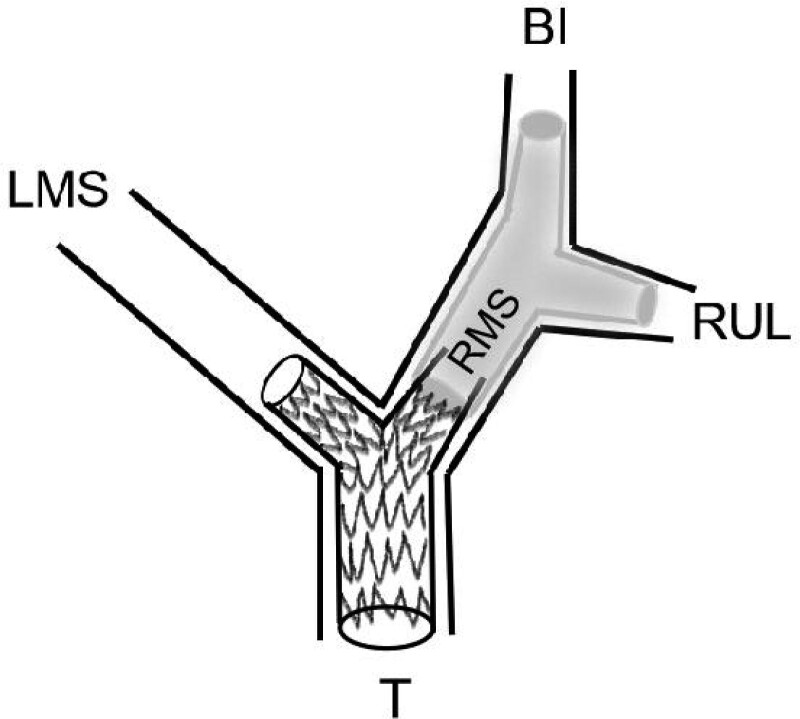
Schematic diagrams of Y-shaped covered metal stent and Y-shaped silicone stent placement. BI = bronchus intermedius; LMS = left mainstem bronchus RUL = right upper lobe; RMS = right mainstem bronchus; T = trachea.

This patient was admitted to the hospital and was diagnosed as MCAO involving trachea carina and left main bronchus, esophagocarinal fistula involving right main bronchus, severe pneumonia, and esophageal cancer. We decided to implant stents under general anesthesia through rigid endoscopy. It was necessary to place permanent stents in the trachea, considering the patient right upper lobe and right middle segment were not obviously invaded, and the esophageal fistula involved the right main bronchus was about 0.5 cm from the right middle segment. The patient right upper lobe function was impaired and right middle segment was easy to form new granulation tissue when the Y-shaped covered metal stent was used alone. When the Y-shaped silicone stent was placed alone, it would be not effective to block the large fistula. Therefore, we designed Y-shaped hybrid silicone stent and placed it in the right main bronchus, and a Y-shaped metal covered stent placed in the lower part of trachea (Fig. 3), which did not only block the fistula, but also relieved the airway obstruction to ensure the function of patient right lung. This patient used high-frequency ventilation during the operation to reduce air leakage and CO_2_ retention. The symptoms of the patient were significantly relieved the next day. Airway stents were unobstructed as confirmed by bronchoscopy.

In conclusion, this case has positively impacted the treatment of patients with similar conditions. Hybrid stent can be used as an alternative in palliative treatment and airway reconstruction of malignant and complex airway diseases to relieves the obstructive symptoms and increases overall patient survival.

## Acknowledgments

The authors express their gratitude to the patient who made this work possible, as well as the professionals and researchers that participated in this study. A patient informed consent was acquired.

## Author contributions

**Methodology:** Mingyao Ke, Yazhi Yong, Bingqing Luo.

**Resources:** Ganzhu Feng.

**Writing – original draft:** Chenghua Zhu.

**Writing – review & editing:** Jingning Liu.
